# Gene Sets for Utilization of Primary and Secondary Nutrition Supplies in the Distal Gut of Endangered Iberian Lynx

**DOI:** 10.1371/journal.pone.0051521

**Published:** 2012-12-12

**Authors:** María Alcaide, Enzo Messina, Michael Richter, Rafael Bargiela, Jörg Peplies, Sharon A. Huws, Charles J. Newbold, Peter N. Golyshin, Miguel A. Simón, Guillermo López, Michail M. Yakimov, Manuel Ferrer

**Affiliations:** 1 Department of Applied Biocatalysis, Spanish National Research Council (CSIC), Institute of Catalysis, Madrid, Spain; 2 Institute for Coastal Marine Environment (IAMC), National Research Council (CNR), Messina, Italy; 3 Ribocon GmbH, Bremen, Germany; 4 Institute of Biological, Environmental and Rural Sciences, Aberystwyth University, Aberystwyth, United Kingdom; 5 Centre for Integrated Research in the Rural Environment, Aberystwyth University-Bangor University Partnership (CIRRE), Aberystwyth, Ceredigion, United Kingdom; 6 School of Biological Sciences, Bangor University, Gwynedd, United Kingdom; 7 Consejería de Medio Ambiente de la Junta de Andalucía, Jaén, Spain; 8 Agencia de Medio Ambiente y Agua de Andalucía, Córdoba, Spain; Wageningen University, The Netherlands

## Abstract

Recent studies have indicated the existence of an extensive trans-genomic trans-mural co-metabolism between gut microbes and animal hosts that is diet-, host phylogeny- and provenance-influenced. Here, we analyzed the biodiversity at the level of small subunit rRNA gene sequence and the metabolic composition of 18 Mbp of consensus metagenome sequences and activity characteristics of bacterial intra-cellular extracts, in wild Iberian lynx (*Lynx pardinus*) fecal samples. Bacterial signatures (14.43% of all of the Firmicutes reads and 6.36% of total reads) related to the uncultured anaerobic commensals *Anaeroplasma* spp., which are typically found in ovine and bovine rumen, were first identified. The lynx gut was further characterized by an over-representation of ‘presumptive’ aquaporin *aqpZ* genes and genes encoding ‘active’ lysosomal-like digestive enzymes that are possibly needed to acquire glycerol, sugars and amino acids from glycoproteins, glyco(amino)lipids, glyco(amino)glycans and nucleoside diphosphate sugars. Lynx gut was highly enriched (28% of the total glycosidases) in genes encoding α-amylase and related enzymes, although it exhibited low rate of enzymatic activity indicative of starch degradation. The preponderance of β-xylosidase activity in protein extracts further suggests lynx gut microbes being most active for the metabolism of β-xylose containing plant N-glycans, although β-xylosidases sequences constituted only 1.5% of total glycosidases. These collective and unique bacterial, genetic and enzymatic activity signatures suggest that the wild lynx gut microbiota not only harbors gene sets underpinning sugar uptake from primary animal tissues (with the monotypic dietary profile of the wild lynx consisting of 80–100% wild rabbits) but also for the hydrolysis of prey-derived plant biomass. Although, the present investigation corresponds to a single sample and some of the statements should be considered qualitative, the data most likely suggests a tighter, more coordinated and complex evolutionary and nutritional ecology scenario of carnivore gut microbial communities than has been previously assumed.

## Introduction

The Iberian lynx (*Lynx pardinus*) is native to the Iberian Peninsula [1] and is considered the most endangered felid species in the world [2]. Iberian lynxes are confined to two isolated populations in southern Spain in the Doñana-Aljarafe and Sierra Morena areas, and only 88 and 224 individuals, respectively, are estimated to remain [3]. To save this species from extinction, an EU LIFE Nature project is underway that includes habitat preservation, lynx population monitoring, and rabbit population management [4]. Additionally, cryopreservation of lynx genetic material and a captive *ex situ* breeding project were initiated to preserve the genetic diversity of the species and to produce new specimens for future reintroduction [5], [6].

At present, this species is critically endangered due to the decline of its basic prey (the wild European rabbit, *Oryctolagus cuniculus*), an increase in non-natural mortality, the fragmentation and loss of its habitat [7], [8] and the effects of infectious diseases [9], [10]. The identification of heavy metals in the tissues of some lynxes [11] also suggests that anthropogenic activities (such as mining) that do contaminate their water reservoirs in Southern Spain (Doñana and Sierra Morena), may impact lynx health and survival. Comparative analysis of the gestagens and the estrogen levels in four lynx species has indicated the possible role of qualitative and quantitative variations in gut bacteria composition as determinants of specific life stages [12]. This idea is consistent with the fact that gut harbors a vast ensemble of microbes that perform vital processes for host physiology and nutrition [13].

Owing to metagenomic approaches, our knowledge of the abundance, diversity and evolution of gut microbes has been fundamentally advanced over the past decade. Fecal microbial communities of approximately 200 animals representing approximately 60 species have been analyzed to date, including human [13], buffalo [14], [15], bovine rumen [16], [17], pig [18], [19], rat [20], turkey [21], swine [22], giant panda [23], wallaby [24], feline [25], canine [26], [27], wild gorilla [28] and wild wolf [29] gut communities, to cite the most significant cases. According to a recent study [13], the diet and phylogeny of a host strongly shape gut bacterial diversity, which increases from carnivory to omnivory and to herbivory, and these bacterial communities co-diversify with their host. The study also indicated that gut bacterial communities from hosts of the same taxa with similar diets are the most similar to each other, regardless of geographic location, and that the capacity to survive largely on a plant-based diet was likely acquired independently. However, no results explain how prey with both monotypic and varied diets may shape the intestinal microbiota of a predator. In this context, the rapidly growing field of gene-centric metagenomic analysis is promoting our understanding of the functions of gut microbial populations. However, the few examples applied to date are restricted to herbivores such as swine [22], Tammar wallabies [24], cows [16], buffalo [15] and giant pandas [23], omnivores such as humans [30] and, to a lesser extent, carnivores such as canines [27]. The results of these studies emphasize that: (i) animal survival may highly depend on gut microbes; (ii) in many cases, the repertoire and diversity of specific gene sets for a particular activity, such as plant biomass conversion, could not be expected from the diet of the animal [24]; and (iii) gene signatures may also be used as pathogenic and antibiotic resistance indicators [22].

Despite extensive information concerning the field ecology of the wild Iberian lynx [9], [31]–[36], to date, no information is available regarding its gut microbial composition, structure and function. Herein, an extensive comparative analysis with respect to microbial composition and functional content has provided the first insight into the presence of metabolic signatures and gene sets enriched in the lynx gut. We also measured enzymatic activities in bacterial intra-cellular enzyme extracts isolated from lynx fecal samples and compared them with those present in rumen content. Our objective was to verify the correlation between the presence and/or occurrence of particular gene sets and their functional performance and to differentiate lynx gut from ruminant communities from a functional point of view. Contrary to previous thought, this study suggests an occurrence of high metabolic complexity in the gut of the wild Iberian lynx and possibly also in other carnivores. It should be noted that our investigation was restricted to the analysis of fecal samples from one-adult wild Iberian lynx.

## Methods

Fresh fecal samples were collected and immediately processed from one-adult wild Iberian lynx from Sierra Morena (Santa Elena, Jaén, Spain; 38°20′″N 3°32′″O) captured as part of the routinely radio-monitoring program of the LIFE-Nature conservation project. Capture was made with a double-entrance, electro-welded-mesh box-trap following international safely standards (ISO 10990-5), and according to the animal welfare specifications of the permit SGYB/FOA/AFR/CFS of the Andalusia Regional Government of Environment. Handling was performed under anesthesia and the individual was safely released after 1 h of capture. The animal, named Eva (weight: 11.1 kg; year of born: 2008), had a normal health status, and the sample was taken 8 hours from the last meal. The corporal status was good and healthy. Fecal DNA was extracted using the MoBio Kit according to the manufacturer’s instructions using 12 g of fecal sample. The quality of the total DNA was checked by agarose electrophoresis and was spectrophotometrically quantified, which indicated a total amount of approximately 20 µg. Functional analysis were also performed using fecal samples of a second animal, named Granadilla (weight: 87.0 kg; year of born: 2010), captured and processed as for the animal named Eva.

### DNA Sequencing, Assembly, Gene Prediction and Annotation

Sequencing was performed with a Roche 454 GS FLX Ti sequencer (454 Life Sciences, Branford, CT, USA) at Lifesequencing S.L. (Valencia, Spain), with one picotiterplate. Assembly was performed with Roche Newbler assembler v. 2.5.3 using the default parameters.

Potential protein-coding genes were identified with MetaGene [37]. Additionally, contigs without open reading frame (ORF) predictions by MetaGene were translated into artificial reading frames and were BLAST-searched against the NCBI-nr database for similar sequences. Artificial translations with similarities were further processed in the same manner as the predicted ORFs from MetaGene. Transfer RNA genes were identified with tRNAScan-SE [38], and ribosomal RNA genes were identified with meta-rna 1.0 [39]. Annotation was performed with GenDB, version 2.2 [40], supplemented with the tool JCoast, version 1.6 [41]. For each predicted ORF, observations were collected from similarity searches against the NCBI-nr, Swiss-Prot, Kyoto Encyclopedia of Genes and Genomes (KEGG) and genomesDB sequence databases [41] and against protein family databases from Pfam [42] and InterPro [43]. SignalP was used for signal peptide predictions [44] and TMHMM was used for transmembrane helix analysis [45]. Predicted protein coding sequences were automatically annotated using the in-house software MicHanThi [46]. The MicHanThi software predicts gene functions based on similarity searches using the NCBI-nr (including Swiss-Prot) and InterPro databases. The annotation of proteins highlighted within the scope of this study was the subject of manual inspection. For all observations regarding putative protein functions, an E-value cutoff of 10^−4^ was used.

### Functional Classification with KEGG

To identify potential metabolic pathways, genes were searched for similarity against the KEGG database. A match was considered valid if the similarity search resulted in an expectation E-value less than 1e^−05^ [47]. All occurring KO (KEGG Orthology) numbers were mapped against KEGG pathway functional hierarchies and were statistically analyzed.

### Functional Classification with Cluster of Orthologous Groups (COG)

All predicted ORFs were also examined for similarity against the COG database [48]. A match was considered valid if the similarity search resulted in an E-value less than 1e^−05^.

### Comparison with other Fecal Metagenomes Based on KEGG

An additional set of 23 metagenomes describing animal-associated (mammals and arthropoda) microbiomes for comparative genomics were obtained by the IMG/M webpage of the US Department of Energy Joint Genome Institute (http://www.jgi.doe.gov/) [49]. For each metagenome, the functional classification by KEGG has been downloaded. All of the downloaded lists were parsed by counting matches to one of the 20590 KO entries in the original KEGG hierarchy. The final list was normalized by calculating the percentages of occurrence. This list was used to build heat maps with JHeatChart (http://www.javaheatmap.com). Additionally, a distance matrix was calculated between all of the metagenome KEGG pathways listed by measuring the dependence between the two lists with the Pearson product-moment correlation coefficient. This distance matrix was used to generate the tree and was finally integrated into the heat map.

### In-depth Small Subunit (SSU) rRNA Analysis

Unassembled sequence reads from metagenome sequencing were pre-processed (quality control and alignment) by the bioinformatics pipeline of the SILVA project [50]. Briefly, reads shorter than 200 nucleotides and with more than 2% ambiguities or 2% homopolymers were removed. The remaining reads were aligned against the SSU rRNA seed of the SILVA database release 106 (http://www.arb-silva.de/documentation/background/release-106), whereupon non-aligned reads were not considered for downstream analysis. Using this strategy, putative partial SSU rRNA gene reads within the dataset could be extracted. Subsequently, remaining reads were (1) dereplicated, (2) clustered, and (3) classified. For statistical reasons, the considerable overlapping of extracted reads in terms of gene region coverage is extremely unlikely; therefore, steps (1) and (2) cannot lead to any meaningful information. However, from a technical perspective, the procedures are favorable because they minimize the number of reads in the final classification step. Dereplication (the identification of identical reads) was performed with cd-hit-est of the cd-hit package 3.1.2 (http://www.bioinformatics.org/cd-hit) using an identity criterion of 1.00 and a word size of 8. The remaining sequences were clustered again with cd-hit-est using an identity criterion of 0.98 (same word size). The longest read of each cluster was used as a reference for taxonomic classification, which was performed by a local BLAST search against the SILVA SSURef 106 NR dataset (http://www.arb-silva.de/projects/ssu-ref-nr/) using blast −2.2.22+ (http://blast.ncbi.nlm.nih.gov/Blast.cgi) with standard settings. The full SILVA taxonomic path of the best blast hit was assigned to the reads in the event that the value for (% sequence identity+% alignment coverage)/2 was at least 93.0. In the final step, the taxonomic path of each cluster reference read was mapped to the additional reads within the corresponding cluster and the corresponding replicates, which were identified in the previous analysis step, to finally obtain (semi-)quantitative information concerning the number of individual reads representing a taxonomic path.

The presence of archaeal 16S rRNA gene signatures was checked by using the archaeal-specific primers Ar20F (TTCCGGTTGATCCYGCCRG) and Ar958R (YCCGGGGTTGAMTCCAATT) and total DNA as template. Amplification was done in a 20 µl reaction volume with recombinant *Taq* DNA Polymerase (Invitrogen, Germany) and original reagents, according to the PCR protocols, with the annealing temperature of 45°C and 50°C (bacterial and archaeal rRNA, respectively), for 30 cycles.

### MIxS Submission

Consistent contextual data acquisition for MIxS- compliant submission by using the environmental package ‘host-associated’ has been done using JCoast v1.7.

### Phylogenetic Analysis of SSU rRNA Genes

For the SSU rRNA gene sequences, initial alignment of amplified sequences and close relatives identified with BLAST [51] were performed using the SILVA alignment tool [50] and manually inserted in ARB [52]. After alignment, the neighbour-joining algorithm of ARB program package was used to generate the phylogenetic trees based on distance analysis for SSU rRNA. The robustness of inferred topologies was tested by bootstrap re-sampling using the same distance model (1,000 replicates).

### Preparation of Protein Crude Extracts and Enzyme Assays

Fresh fecal samples collected from two-adult wild Iberian lynxes, named Eva (for which DNA was isolated and analyzed here) and Granadilla, were used for activity test. To prepare enzyme extracts, fresh fecal samples (5.00 and 3.97 grams for Lynx named Eva and Granadilla, respectively) were firstly homogenized with 50 ml (for Eva) and 15 ml (for Granadilla) buffer solution (5 mM sodium pyrophosphate, pH 7.8; Sigma Chemical Co., St. Louis, MO, USA) containing Tween® 80 (final concentration, 1 mg liter^−1^; Sigma Chemical Co.) to facilitate microbial distribution. For microbial dispersion from fecal samples, the solution was sonicated on ice in an ultrasonic cleaner (Bandelin SONOREX RK31; Bandelin electconic, Berlin, Germany) with the amplitude set at 100% of the maximum. The dispersion time for samples was 90 min. After dispersion, the samples were then centrifuged at 9 *g* at 4°C for 10 minutes to remove fecal debris and the cell pellet was used directly for protein extraction. The supernatant was then centrifuged at 8,500 *g* for 10 min at 4°C to pellet the microbial cells which were kept at −80°C until use.

Rumen contents were collected from four rumen-fistulated, non-lactating Holstein cows (average weight of 731 kg) housed at Trawsgoed experimental farm (Aberystwyth, Ceredigion, Wales). Samples were retrieved under the authorities of the UK Animal (Scientific Procedures) Act (1986). Animals were fed a diet composed of a mixture of grass silage and straw (75∶25) *ad libitum*. Sampling was carried out 2 h after the morning feed. Rumen samples were harvested and then processed to produce two fractions: strained ruminal fluid (SRF) and liquid-attached bacteria (LAB). For SRF retrieval, total ruminal content was strained through four layers of muslin in order to remove large particles, and SRF was then frozen at −80°C until use. Cell pellet was obtained using the same protocol as for the Lynx (Granadilla) fecal samples (see above) using the total amount (5.09 g) of ruminal material. For LAB retrieval, approximately 1 liter of mixed total rumen content was hand squeezed to get rumen liquor, and the solid fraction was put in a large foil tray. The liquid fraction was spun at 2,000 *g*, 10 min, 4°C (MSE Europa 24 M, Berthold Hermle KG, Weisbaden, Germany); the supernatant was then strained through a 1 mm^2^ pore-sized nylon mesh to remove feed particles, and spun again at 13,000 *g*, 25 min, 4°C. The pellet was washed in a saline solution (made from 180 g NaCl dissolved in 20 L distilled water), and subsequently centrifuged at 13,000 *g*, 25 min, 4°C. The pellet was re-washed with distilled water, and spun down at 13,000 *g*, 15 min, 4°C. The pellet, containing LAB, was then transferred into a sterile jar and kept at −80°C until use. Cell pellet was obtained using the same protocol as described above for lynx fecal samples using the total amount (430 mg) of material and 3 ml Tween® 80-supplemented sodium pyrophosphate solution.

Protein extraction (for both lynx feces and rumen samples) was performed by incubating 10 ml (for Eva), 15 ml (for Granadilla and SRF) and 4 ml (for LAB) 4-(2-hydroxyethyl)piperazine-1-ethanesulfonic acid (HEPES) buffer (40 mM, pH 7.0) supplemented with 3 µl Lysonase™ Bioprocessing Reagent (Novagen) for 60 min at room temperature with the microbial pellet obtained as described above. Fecal or ruminal bacteria were further disrupted by mechanical lysis followed by sonication for 2.5 min on ice using an ultrasonicator equipped with a 3-mm tapered microtip and with the amplitude set at 10-W. The extract was then centrifuged at 4°C for 10 min at 12,000 *g* to separate cell debris and intact cells. The supernatant was carefully aspirated (to avoid disturbing the pellet), transferred to a new tube and freeze dried. Dry material was stored at −20°C until use and re-suspended in buffer 20 mM HEPES, pH 7.0, prior to use. Using this protocol the total amount of proteins recovered for the four samples were: 12.5 mg per 5.00 gram of fecal sample of the Lynx named Eva, 8.1 mg per 3.97 gram of fecal sample of the Lynx named Granadilla, 20.00 mg per 5.09 gram of ruminal SRF material and 21.00 mg per 430 mg of fecal sample of LAB rumen content.

Enzymatic activity was quantified in 96-well plates using a BioTek Synergy HT spectrophotometer by measuring release of *p*-nitrophenol (*p*NP) using a protein amount of 6.34 µg (for Eva), 7.74 µg (for Granadilla), 15.83 µg (for SRF) and 15.42 µg (for LAB), and [substrate] of 1 mg ml^−1^ (from a 10 mg ml^−1^ stock solution) in 20 mM glycine buffer, pH 9.0, *T* = 30°C, in a final volume of 50 µl. Enzyme and control tests were incubated for 5 to 960 min for assays. Under our experimental conditions, the absorption coefficient for *p*NP was measured as 15,200 M^−1^·cm^−1^. In all cases, one unit (U) of enzyme activity was defined as the amount of protein producing 1 µmol of reducing sugars in 1 min under the assay conditions. Unless otherwise stated, all assays were performed using technical replicates. The following substrates (all from Sigma Chemical Co., St. Louis, MO, USA) were used for activity tests: *p*NP-α-glucopyranoside, *p*NP-β-D-glucopyranoside, *p*NP-α-maltoside, *p*NP-α-D-maltopentaoside, *p*NP-α-D-maltohexaoside, *p*NP-β-D-cellobioside, *p*NP-α-L-galactopyranoside, *p*NP-β-D-galactopyranoside, *p*NP-α-xylopyranoside, *p*NP-β-xylopyranoside, *p*NP-α-arabinopyranoside, *p*NP-β-arabinopyranoside, *p*NP-α-arabinofuranoside, *p*NP-α-L-rhamnopyranoside, *p*NP-α-mannopyranoside, *p*NP-β-D-mannopyranoside, *p*NP-β-lactopyranoside, *p*NP-β-lactoside, *p*NP-α-fucoside, *p*NP-β-fucoside, *p*NP-β-glucuronide and *p*NP-β-acetylglucuronide.

### Deposition of Sequence Data

Project has been registered as umbrella BioProject at NCBI with the ID PRJNA158313. This Whole Genome Shotgun project has been deposited at DDBJ/EMBL/GenBank under the accession AMCI00000000. The version described in this paper is the first version, AMCI01000000.

## Results and Discussion

### General Comments

Recent research has disclosed tight connections between host diet and microbiome phylogenetic and metabolic diversity [13]. In this context, as wild lynx is well known to have a monotypic diet that consists primarily of wild European rabbits, it could be used as a model to interrogate how a specific prey may shape predator intestinal microbiome. This is of a special significance, as the microbiome analysis of a carnivore with monotypic prey diet has not been yet reported, which may reflect the genetic potential within an ecosystem. Following on from this, the analysis of both microbial diversity and microbial genomic and functional contents in fecal samples of a healthy adult lynx was undertaken. Instead of performing an extensive phylogenetic analysis, special emphasis was given to the identification and analysis of gene signatures and their associated activities present in the gut microbiome.

To uncover the genomic information of the dominant colonic bacteria in wild lynxes, fecal samples were collected from a healthy adult lynx captured near Santa Elena (Jaén, Spain) (Figure S1). The extracted DNA was directly pyrosequenced using a Roche GS FLX DNA sequencer, which produced 795,151 reads with an average length per read of 366.7 bp and a total of 291.59 Mbp of raw DNA sequences. Circa 146,153 reads (or 18.38% total reads) were singletons, i.e., reads that were not assembled into contigs; singletons longer that 100 nucleotides accounted 140,429 (or 17.67%).

### General Features of the Microbial Diversity in the Distal gut of the Wild Lynx

**Figure 1:**
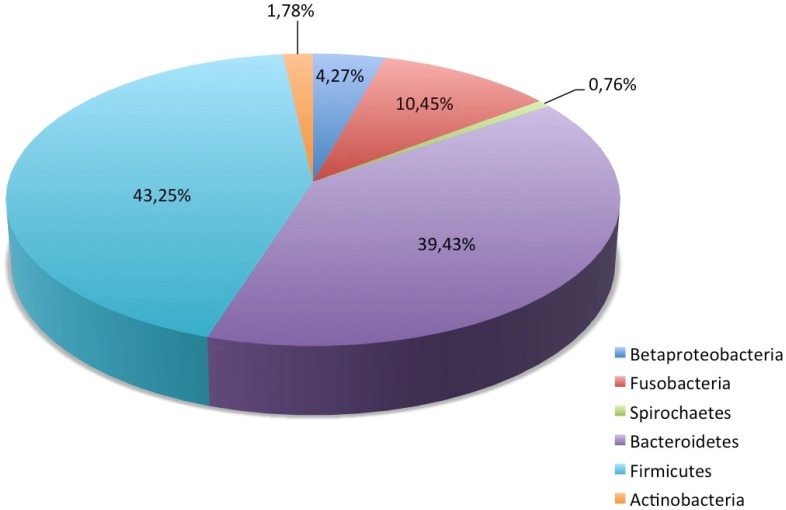
Overview of the relative abundance of phylogenetic prokaryotic groups recovered from the lynx distal gut based on SSU rRNA tag sequences extracted from the pyrosequences. All reads belonging to phylum Proteobacteria identified related to Betaproteobacteria, which is only cited in the Figure.

The biodiversity at the level of small subunit (SSU) rRNA was analyzed on the online server using SILVA databases. For this purpose, we just used those raw (unassembled) sequences with a length >200 nucleotides obtained after direct pyrosequencing of the extracted DNA (Figure S2). All shorter sequences were discarded. As result, 1,570 reads (or 0.2% of all of the total reads) found matches in this database. The ensemble of SSU rRNA sequences produced in this study provides an overarching, although incomplete, view of the lynx gut microbiota. All of the recovered sequences were unambiguously assigned; they affiliated exclusively with the kingdom Bacteria, with the absolute dominance of the three phylogenetic lineages of Firmicutes (43.25%), Bacteroidetes (39.43%) and Fusobacteria (10.45%) (Figures 1 and S3). Members of Proteo- and Actinobacteria were also identified in relatively abundant quantities (4.27 and 1.78%, respectively), whereas the phylum Spirochaetes was represented by 12 reads that accounted for only 0.76% of the total SSU sequences. Candidate division TM7, which is a recently recognized major lineage of the domain Bacteria with unknown cultivated representatives, was recovered from the distal gut of the wild lynx as singleton.

As shown in Figure S4, more than two-fifths of all of the reads (679 out of 1,570) affiliated with the phylum Firmicutes represented 11 different families of the following five classes: Clostridia, Erysipelotrichi, Negativicutes, Mollicutes and Bacilli. Lachnospiraceae and Ruminococcaceae were the largest clostridia families and accounted for almost half of all of the reads belonging to phylum Firmicutes (186 and 120 reads out of 679, respectively). Although mammalian gut microbes are highly adapted to their habitats, many lineages are extremely rare outside of mammalian guts and resist cultivation [53]. Accordingly, only a G6Y_G13M sequence was related to a cultivated organism, *Howardella ureilytica* (DQ925472), whereas the other reads were closely related to the uncultivated members of gut microbiota recovered from various carnivorous mammals [13]. The members of three additional families of class Clostridia, namely Peptostreptococcaceae, Clostridiaceae and representatives of Family XIII *Incertae sedis,* were present in lynx gut microbiota at varying frequencies (9.43%, 1.18% and 2.8% of all Firmicutes reads, respectively; Figure S5). More than 140 reads, which accounted for almost 21% of all Firmicutes reads, fell into class Erysipelotrichi and were unambiguously classified within Erysipelotrichaceaei. Aside from 28 identical reads belonging to *Allobaculum stercoricanis* (canine feces isolate), no relation to other cultivated representatives of this family was noted. Class Negativicutes was represented by the family Veillonellaceae and contained 24 reads (3.53% of all of the Firmicutes reads) that were closely related to uncultivated bacterium recovered from human feces (DQ824172). Of the different Firmicutes-related phylotypes recovered from lynx feces, almost 100 identical reads were somewhat similar to an uncultured *Anaeroplasma* sp. clone EMP_L44 (EU794312) that was found in cattle fecal microbiota (Figure S5). The identification of microbes related to Anaeroplasmatales of class Mollicutes (according to ARB, SILVA affiliated this class with the phylum Firmicutes) was unexpected because members of this order are known as anaerobic commensals that are typically found in the rumen [54]. The intestinal material from a ruminant-like animal such as a rabbit that was eaten by the lynx could be considered a possible source of these microbes in the lynx fecal microbiota. Because it has been demonstrated that *Anaeroplasmas* spp. play important roles as pathogens of different hosts [55], progress in understanding the relationship between commensal *Anaeroplasma* spp. and lynx health is likely to promote the identification of new parasitic events derived from ruminant-like *Anaeroplasma* spp. [56].

The phylum Bacteroidetes was represented by 619 reads (39.43% of all reads) belonging to three families: Bacteroidaceae, Prevotellaceae and Porphyromonadaceae (Figure S6). Aside from reads affiliated with the genera *Prevotella*, *Paraprevotella* and *Odoribacter*, members of this group were similar to generally unclassified bacteria recovered from the gut microbiota of different animals and birds [57]. A total of 164 reads that represented six different subgroups were identified within the phylum Fusobacteria (Figure S6); more than 90% of these reads were taxonomically affiliated with the members of the family Fusobacteria, among which 110 reads were found be very similar to *Fusobacterium perfoetens* (M58684). The diversity of the only 67 reads belonging to Proteobacteria identified was extremely low, with all of the reads placed within the genus *Sutterella* of Alcaligenaceae. In contrast, a total of 28 reads representing seven subclusters were identified within the phylum Actinobacteria (Figure S6). This phylum was mainly represented by members of the family Coriobacteriaceae (almost 93% of actinobacterial reads). Most of the Coriobacteriaceae reads were classified into the genera *Collinsella* and *Slackia* and showed >98% similarity to *Collinsella intestinalis* (AB031063) and *S. faecicanis* (AJ608686). Noticeably, 12 reads recovered from the lynx metagenome were affiliated with the phylum Spirochaetes, 10 (or >83%) of which are organized into a cluster closely related to the SSU rRNA gene sequences recovered from the feces of omnivorous primates (EU778629) [13]. To our knowledge, this result is the first indication of the presence of this bacterial group in the fecal microbiota of carnivorous mammals. No reads related to SSU rRNA genes of Archaea were detected in the lynx fecal metagenomic pyrosequencing data; however, standard PCR survey revealed the presence of archaeal SSU RNA gene signatures (not shown). This finding contrast with the PCR-based analysis performed on the fecal microbiota of wolves and other carnivorous mammals [29], [57], where no indication of archaeal genes was found. Further studies will be required for the characterization of the archaeal community populating the distal gut of wild lynx.

### The Sequencing and Gene Prediction of Lynx Distal Gut Microbiota

DNA sequences were assembled into an 18.34 Mbp sequence with an average GC content of approximately 47.74% (11,068 contigs, of which 8,084 were longer than 500 bp; N_50_ of 4,370 bp and longer contig of 68,169 bp). From the meta-sequence, we identified 23,780 potential protein-coding genes (cut-off of ≥ 20 amino acid-long sequences). Even when suspicious hypothetical open reading frames (ORFs) ≥ 150 bp were excluded, 29.7% (or 7,052 hits) of the protein sequences deduced were hypothetical proteins that did not exhibit any sequence similarity to known proteins in public databases. Another 22.1% (or 5,263 hits) of the protein sequences exhibited similarity to proteins of unknown function (conserved hypothetical proteins). Thus, a substantial fraction of the genes found in the lynx gut were entirely novel with as yet unknown functions. Of the non-hypothetical genes (11,465 or 48.2%), 84.1% could be assigned to a total of 10,587 Clusters of Orthologous Groups (COGs), and 66.7% could be assigned to KEGG) pathways (Table S1). On average, 2.5 genes belonged to each COG. The taxonomic level of the gene catalogue with the associated protein sequences was determined by BLASTP analysis (for details see Methods). Approximately 30% (or 6,625 out of 23,780) of lynx gut sequences could not be assigned to any characterized microbe, whereas the remaining sequences could be assigned to approximately 287 particular genera. Although these data may be overestimations, the taxonomic distribution of assigned genes is comparable to that determined by SSU rRNA assignment (see Table S2 for detailed information regarding metagenome annotation).

### The Lynx Gut Microbiome Possesses Gene Sets for the Efficient Metabolism of Animal Tissues

Functional assignment of the predicted genes (23,780 in total) was made on the basis of BLASTP analysis against a reference dataset for COG and KEGG assignments (see Methods). In addition, to compare the overall sequence similarities among the microbiome of lynx gut and other 23 animal-associated gut microbiomes (for details see Table S3), we performed a reciprocal BLASTP analysis of the entire gene set for each microbiome, followed by MDS clustering against the normalized distance matrix (see “Methods”), that overall avoid artifacts due to differences in sample size (i.e. number of reads and ORFs, to cite some). To predict the metabolic potential and to identify significant over- and under-represented COGs and KEGGs, the JCoast annotation pipeline was used.

**Figure 2:**
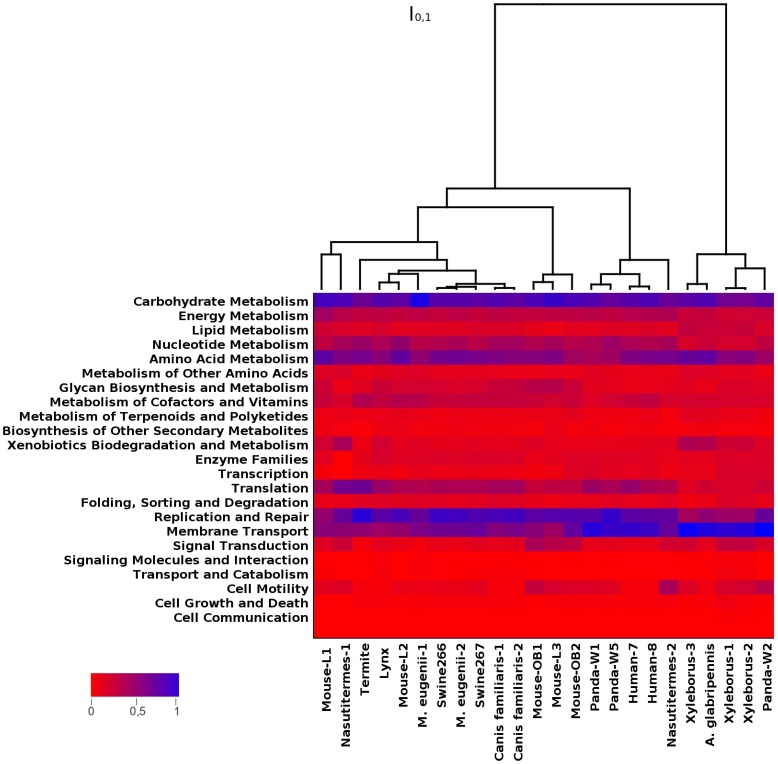
Comparison of lynx with other animal-associated (mammals and arthropoda) gut metagenomes. Clustering of gut metagenomes available within IMG/M based on functional composition of KEGGs.

Profiling analysis based on the KEGG enrichment values that was calculated for each microbiome (Figure 2) showed overall similar KEGG distributions for all of the gut microbiomes examined, with an over-representation of KEGGs classified into the ‘Carbohydrate Metabolism’, ‘Replication and Repair’, ‘Amino Acid Metabolism’ and ‘Translation’ categories and an under-representation of KEGGs included in ‘Cellular Processes’. However, clear differences were observed that characterized the lynx gut, including significant over-representation of KEGGs for ‘Signaling Molecules and Interaction’ and ‘Transport and Catabolism’ and the remarkable under-representation of ‘Cell Motility’, which was unique for the lynx gut (Table S3). The heat map and clustering analysis shown in Figure 2 further suggested that the lynx gut microbiome was functionally closer to the microbiomes of guts from other mammals, such as mice, swine, marsupials (*Macropus eugenii*) and canines (*Canis familiaris*). Moreover, the lynx gut metagenome clusters more closely with the termite metagenome than with panda and human gut metagenomes.

**Figure 3:**
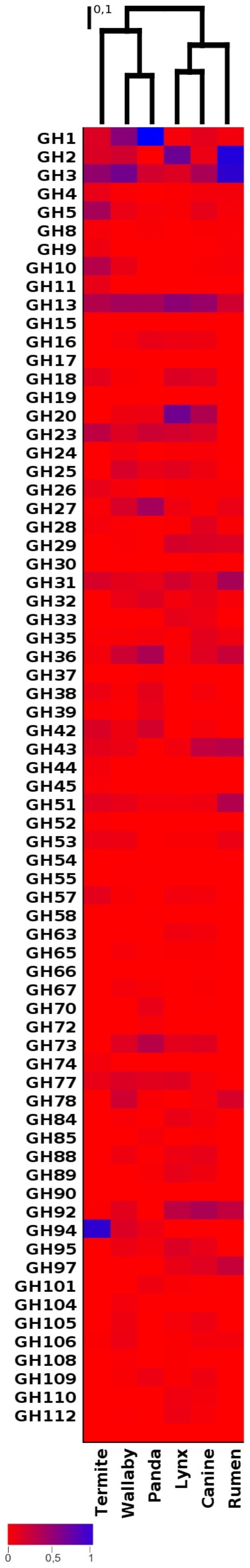
Heat map and clustering of predicted genes encoding GHs in the lynx, termite, bovine rumen, panda, wallaby and canine gastrointestinal metagenomes. The heat map colors represent the relative percentages of GH families within each sample. Hierarchical clustering based on relative percentage of predicted genes encoding GHs is specifically shown.

The striking depletion of ‘presumptive’ genes for the biosynthesis of flagella and for chemotaxis (only 15 genes identified: namely, 4 *che*AYR, 3 *mot*B, 2 *flg*J, 5 *pil*BFQT and 1 *cpa*F genes) within the ‘Cell Motility’ category was noteworthy (Table S3). This KEGG category accounts for only approximately 0.66% of the total KEGGs, whereas the average value for the 23 gut microbiomes considered herein was approximately four-fold higher (Table S3). Recently, it was proposed that motility could be considered advantageous for intestinal microbes because it facilitates access to easily digestible food sources [58]. The fact that the lynx diet consists of a high-energy animal tissue diet (80–100% of which consists of rabbits) containing a low amount of free carbohydrates (approximately 1.2%) may explain why flagellin genes are unnecessary. Additionally, as it has been suggested for microbes operating in the adult human gut, it is possible that the abnegation of motility is an adaptation mechanism for gut microbes to be able to persist in the intestinal environment because flagella are highly immunogenic and may lead to the bacteria being discarded [59].

The putative over-representation in lynx gut (circa two-fold) of genes for ‘Signaling Molecules and Interaction’ (0.57% *versus* 0.27% average values) and ‘Transport and Catabolism’ (0.77% *versus* 0.34% average values) KEGG categories (Table S3) was a result of the prevalence of gene signatures encoding proteins that were ‘presumptively’ involved in the metabolism and uptake of fatty glycerols, glycoproteins, glyco(amino)lipids or glyco(amino)glycans and nucleoside diphosphate sugars (Table S4), that will be discussed below.

Putative aquaporin AqpZ was the first group of proteins that was over-represented in lynx gut. These proteins are highly efficient water/glycerol channels that function at high rates in the gastrointestinal system [60]. Eight distinct AqpZ proteins (out of 12,725 assigned to KEGG pathways) were identified in the lynx pyrosequences (Table S4), with six and two sequences binned to several genomes of the Bacteroidetes and Firmicutes phylum, respectively. Representatives of this protein family were also found, although to lesser extents, in the gut microbiomes of larvae *Anoplophora glabripennis* (0.014% total ORF), the ambrosia beetle *Xyleborus affinis* (0.013% total ORF), the marsupial *Macropus eugenii* (0.007% total ORF) [24], swine (0.005% total ORF) [22] and *Canis familiaris* (0.0012% total ORF) [27], as compared to lynx gut (0.034% total ORFs) (Table S3).

The second group of over-represented proteins that contributed to the ‘Signaling Molecules and Interaction’ and ‘Transport and Catabolism’ KEGG categories included ‘presumptive’ NEU1 sialidases (K01186), hyaluronoglucosaminidases (HYA; K01197), α-galactosidases (GLA; K01189), α-N-acetylglucosaminidases (NAGLU; K01205), N-acetylgalactosamine-6-sulfatases (GALNS; K01132), N-acetylglucosamine-6-sulfatases (GNS; K01137), iduronate 2-sulfatases (IDS; K01136) and arylsulfatases (ARSA; K01134), which accounted for almost 0.25% of all ORF (Tables S3 and S4). Only mammal (but not insect) guts were found to contain some of these proteins, although to lesser extents compared to the lynx gut and never possessing this entire set, with the *Canis familiaris* gut containing the highest number of these genes (0.031% of all ORF; Table S3). Acid GLA α-galactosidases (15 hits) hydrolyze the terminal α-galactosyl moieties from complex glycolipids and glycoproteins such as ceramide trihexoside [61]; sialidases (8 hits) help break down large sugar molecules (oligosaccharides) attached to certain glyco-proteins by removing sialic acid [62]; HYA hyalurono-glucosaminidases (7 hits) catalyze the hydrolysis of hyaluronic acid and similar glycosaminoglycans that are found in numerous animal tissues, such as joints, cartilage, skin and eyes [63]; NAGLU (8 hits), GALNS (7 hits), GNS (5 hits), ARSA (7 hits) and IDS (2 hits) may contribute to the putative hydrolysis of the *N*-acetylgalactosamine (GalNAc) and *N*-acetylglucosamine (GlcNAc) components of animal tissues [64]–[66]. Binning analysis suggested that all but one of this second set of sequences binned to several genomes derived from bacteria belonging to the Bacteroidetes phylum, most likely of the *Bacteroides* genus (Table S4).

The third group of over-represented proteins contributing to the ‘Signaling Molecules and Interaction’ and ‘Transport and Catabolism’ KEGG categories included ‘presumptive’ β-galactosamide-α-2,3-sialyltransferases (K00785; 6 hits), β-phosphoglucomutases (or vacuolar sugar phosphorylases) (K01838; 14 hits) and NagZ β-N-acetylhexosaminidases (K01207; 50 hits) (Table S4), which were found to be abundant in the lynx gut (0.29% of all ORF) but were practically absent in other intestinal environments (Table S3). All but three of the third set of sequences most likely binned to several genomes derived from bacteria belonging to the Bacteroidetes phylum (Table S4).

In addition, an over-representation of ‘presumptive’ 5′-nucleotidases in the lynx gut was also evident (K01081; 13 hits or 0.054% of all ORF), which was also observed in mammal (but not insect) gut metagenomes (Table S3). It may be that 5′-nucleotidases provide a complete system for the hydrolysis of extracellular nucleoside diphosphate sugars (activated sugar forms attached to glycoproteins and glycolipids in animal tissues) to nucleoside and non-phosphorylated sugar that can be easily transported into the cells and used as a carbon source [67]. We finally identified ‘presumptive’ prolyl endopeptidases that included one lysosomal Pro-X carboxypeptidases (PRCP; K01285) and 22 prolyl dipeptidyl peptidases (DDP4; K01248), possibly linked to the metabolism of peptides. All but one sequences of the nucleotidases and endopeptidases binned to several genomes derived from bacteria belonging to the Bacteroidetes phylum (Table S4).

### The Lynx Gut Microbiome also Possesses Gene Sets for the Hydrolysis of Plant/Seed Biomass

All the above data confirmed the ‘presumptive’ potential of the lynx gut microbial community for sugar uptake from animal tissues, in agreement with the fact that approximately 50% of all proteins in tissues are glycosylated, with the predominant sugars being glucose (Glc), galactose (Gal), mannose (Man), fucose (Fuc), *N*-acetylgalactosamine (GalNAc), *N*-acetylglucosamine (GlcNAc) and *N*- sialic acid (Sia). We further investigated the presence of additional signatures for sugar metabolism. Functional assignment of predicted genes encoding glycosyl hydrolases (GHs) and carbohydrate-binding modules (CBMs) was performed via BLASTP analysis against the Carbohydrate Active Enzymes (CAZy) database [68]. All hits with an E-value of less than e^−05^ and sequence homology ≥50% were considered and manually analyzed. As a result, out of 23,780 sequences, 372 CAZy-like proteins were identified (Table S4). To predict metabolic potential and to identify significant over- and under-represented GH genes a cluster analysis was performed that considered both the diversity and the relative abundance of GH genes. The GH profile clustering of the lynx, termite, bovine rumen, giant panda, wallaby and canine is shown in Figure 3, which further evidenced that in terms of carbohydrate-related enzymes, the lynx gut microbiome was more closely related to canine gut microbiome and, to a lesser extent, to that of bovine rumina, pandas and wallabies (which cluster together) and termites (Figure 3).

As shown in Table S4, the five most abundant protein families in the lynx gut were putative GHF13 α-amylase and the related GHF57 and GHF77 enzymes (54 sequences), GHF2 β-galactosidase/β-glucuronidase/β-mannosidase and related enzymes (50 sequences), GHF20 lysosomal β-hexosaminidase/lacto-N-biosidase/β-1,6-N-acetylglucosaminidase (48 sequences), GHF92 α-1,2-mannosidase (22 sequences) and GHF23 lysozyme type G/peptidoglycan lyase (15 sequences). Two interesting observations can be made by comparing the relative contributions of these protein families with those found in other gut microbiomes. Among the metagenomes analyzed, the lynx gut microbiome was most enriched in putative GHF13 α-amylase and related enzymes (14.6% of the total GHs); additionally, putative GHF2 β-galactosidase/β-glucuronidase/β-mannosidase and related enzymes represented approximately 13.4% of the total GHs identified in the lynx gut. This abundance is in striking contrast with the identification of only 1.7% of such enzymes in the *Canis familiaris* metagenome (Table S4 and Figure 3). The abundance of these enzymes suggested that starch- and galactomannan-hydrolytic systems for ‘presumptive’ plant/seed cell wall hydrolysis are putatively abundant in the lynx gut community. These findings were entirely unexpected considering the dietary profile of the wild lynx, which predominantly consisted of wild rabbits.

The high number of putative GHF20 proteins (approximately 13% of the total GHs) when compared to canine (7.2% of the total GHs), giant panda (1.6% of the total GHs) and Tammar wallaby (1.4% of the total GHs) microbiomes (Table S4 and Figure 3) was also particularly significant. No representatives of this family of proteins were identified in the termite hindgut and the bovine rumen. Enzymes of this family are known to hydrolyze terminal N-acetyl-D-hexosamine residues in N-acetyl-β-D-hexosaminides found in gangliosides that are abundant animal tissue components. In addition, the metagenome data produces eight sequences (or 2.2% of the total GH) of putative GH89 α-N-acetylglucosaminidases, which may be relevant for the ‘presumptive’ degradation of heparan sulfates and mucopolysaccharides found in all animal tissues [64]. Only the canine and the occasionally carnivorous giant panda guts contain GHF89 proteins (1.4% and 0.5% of the total GHs, respectively) (Table S4). The number of putative α-galactosidase genes (16 sequences in total) identified in the metagenomic dataset was evenly distributed among GHF27, GHF36, GHF97 and GHF110, with representatives of GHF110 only found as lynx- (1.3% of the total GHs) and canine-specific (1.0% of the total GHs) (Table S4). Enzymes of this family are known to hydrolyze terminal, non-reducing α-D-galactose residues in galactose oligosaccharides such as galactolipids, which are also abundant in all animal tissues, and galactomannans. Furthermore, genes matching putative GHF33 lysosomal (trans)sialidases/neuraminidases (2.2% of the total GHs) that hydrolyze glycosidic linkages of terminal sialic residues in glycoproteins and glycolipids were found to be carnivore-specific and slightly over-represented in the lynx gut metagenome compared to that of canine (1.5% of the total GHs; Table S4). Putative GH63 enzymes (1.3% of the total GHs), involved in the formation and hydrolysis of mannosyl-oligosaccharides, likely belong to the same group of carnivore-specific α-glucosidases (Table S4 and Figure 3). Additionally, the lynx metagenome was the most enriched microbiome in putative α-fucosidases GHF29 and GH95 (7.0% of the total GHs) and related enzymes targeting fucose, namely K05305 fucokinases (EC 2.7.1.52; 6 hits), with almost no representatives in other gut microbiomes (Table S4). Fucose is found on N-linked glycans on mammalian, insect and plant cell surfaces but also in bacterial polysaccharides. The under-representation of these enzymes in herbivorous intestines may be likely related to dietary specificity, although this hypothesis must still be clarified.

In contrast to the compared gut microbiomes, few ‘presumptive’ GHF5/GHF28/GHF53 hemicellulases and cellulases (5 hits or 1.3% of the total GH) were found in the lynx gut metagenome (Table S4); in accordance with this observation, the lynx gut also exhibited an under-represented set of GHF1 and GHF3 β-glucosidases and related enzymes (13 hits or 3.8% of the total). Sequences of those families were highly abundant (from 15.6 to 28.0% total hits) in other mammal and insect gut microbiomes (Table S4 and Figure 3).

Binning analysis revealed that 87% of all sequences contributing to the ‘presumptive’ hydrolysis of plant/seed biomass related to several genomes derived from bacteria belonging to Bacteroidetes phylum (most likely from members of the *Bacteroides* genus), 12% to Firmicutes (most likely from members of the *Clostridium* genus) and 1% to Actinobacteria (Table S4). Notably, the contribution of sequences belonging to phylum Firmicutes was much higher to that found for sequences contributing to the ‘presumptive’ sugar uptake from animal tissues (less than 3%).

Taking together, the most notable conclusions drawn from all of these datasets were the presence of unique signatures for GH and associated enzymes ‘presumptively’ linked to sugar uptake from glycoproteins, glyco(amino)lipids and/or glyco(amino)glycans and nucleoside diphosphate sugars most likely present in animal tissues and the virtual absence of cellulases and hemicellulases. Whereas the first observation is consistent with the metabolic capacity previously reported for carnivore (i.e. canine) gut [27], the second one suggests that the lynx gut appears to be an extensive reservoir of genes encoding enzymes attacking the α-glucose, α-mannose and β-galactose side chains of complex plant/seed polysaccharides at proportions much higher than those found in some herbivores (Table S4). Additionally, we further observed that sequences for ‘presumptive’ several ABC-type sugar transport systems, such as the ribose RbsB, methyl-galactoside MglB and maltose/maltodextrin MalE transport systems, were absent in the lynx gut (Tables S2 and S3); an in-depth analysis of sugar transport signatures revealed only a restricted gene set encoding transporters of sugar ABC-like permeases and phosphotransferase (PTS)-like sugar transport proteins, which were found in high numbers in other gut microbiomes (Table S3).

### Detection of Putative Enzyme Signatures for Sugar Metabolism Based on Activity Assays

Microbial ability to thrive in ecological niches depends upon adaptation of their enzymatic machinery to physical-chemical environmental constraints *in situ*. Recently, an association between activity levels of specific transformations and the gene abundance or the abundance of its associated encoding transcripts was reported [69], [70]; however it should be taken into account that enzymes commonly work in complex environments where multiple factors may, all together, affect and modulate their performance independently of their transcript level. Accordingly, using a complementary approach to the analysis of the gene repertoire, we aimed to improve our understanding of lynx-gut bacteria system by examining the sugar-degrading capacity of protein extracts from fecal samples from two wild lynxes, one corresponding to that for which DNA was isolated and analyzed here (named Eva) and a second one, named Granadilla, captured in the same area and under the same protocols. In parallel, protein extracts from rumen content from four rumen-fistulated and non-lactating Holstein cows were isolated and used for comparative purposes. This sample was selected as a model ruminant-like animal because its availability; although, a better model might be to investigate the activity characteristics of the lynx basic prey (the wild European rabbit), technical limitations to capture wild animals (no hunting was allow during the year of lynx sample collection), limits this option. It should be noticed that the assays were restricted to intra-cellular activities present in bacterial cells rather than in the whole fecal samples to avoid the possible inference of host enzymes. Twenty-one different synthetic sugar-based substrates were tested using a colorimetric assay. Preliminary tests confirmed alkaline pH (9.0) as the most convenient for activity determination; to avoid protein instability temperature assay was set at 30°C.

**Figure 4:**
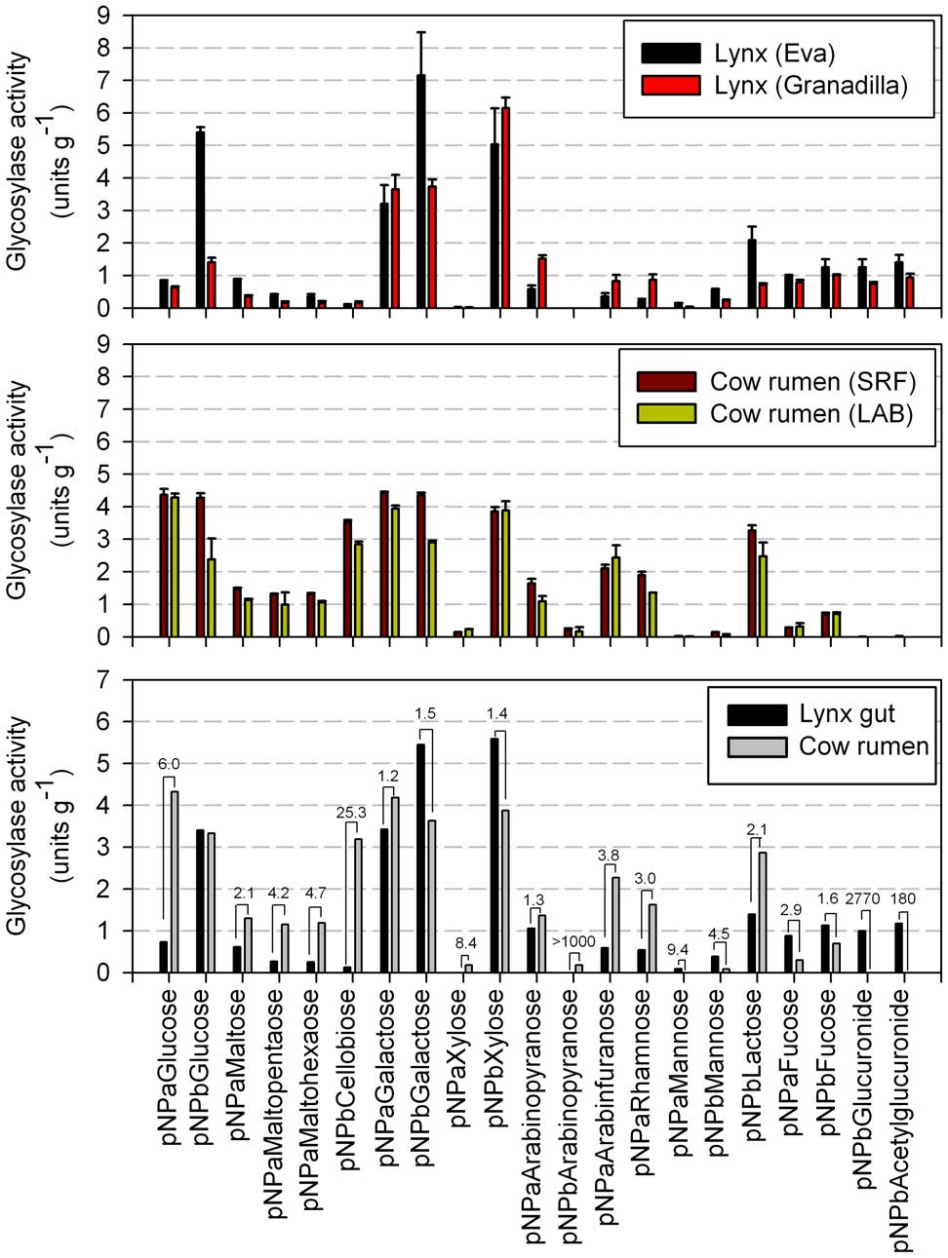
Intracellular enzyme (glycosyl hydrolase) activities associated to bacterial enzyme extracts isolated from two wild Iberian lynx fecal samples and rumen content from four rumen-fistulated and non-lactating Holstein cows. (A) Average potential hydrolysis rates (n = 2, ± standard deviation in three technical replicates) in protein extracts from two wild lynxes (Eva and Granadilla) captured in the same area and under the same protocols. (B) Average potential hydrolysis rates (n = 4, ± standard deviation in three technical replicates) in protein extracts from rumen content from four rumen-fistulated and non-lactating Holstein cows. (C) Comparative average glycosidase activity for lynx gut and cow rumen protein extracts; the fold difference is specifically shown based on data provided in panels A and B. In all cases, enzyme activity was quantified using a BioTek Synergy HT spectrophotometer by measuring release of *p*-nitrophenol (*p*NP) using a protein amount of 6.34 µg (for Eva), 7.74 µg (for Granadilla), 15.83 µg (for SRF) and 15.42 µg (for LAB), and [substrate] of 1 mg ml^−1^ (from a 10 mg ml^−1^ stock solution) in 20 mM glycine buffer, pH 9.0, *T* = 30 °C, in a final volume of 50 µl. The different substrates used as specifically shown. Note: activity against *p*NP derivatives of GalNAc or other mucus-associated sugars could not be determined because they are not commercially available.

As shown in Figure 4, by meaning of specific activity determination (unit g^−1^ protein) we found similar activity profile for the two lynx samples; only major differences were observed when comparing the activity levels for substrates containing β-glucose and β-lactose. GH activities were high, ranging between 7.15±1.32 (for *p*NP-β-galactose) and 0.005±0.0005 (for *p*NP-β-mannose) µmol min^−1^g^−1^ protein. For rumen samples, activities in both strained ruminal fluid (SRF) and liquid-attached bacteria (LAB) from mixed liquid and solid ruminal content were almost indistinguishable from one another, ranging between 4.43±0.032 (for *p*NP-α-galactose) and 0.012±0.001 (for *p*NP-α-mannose) µmol min^−1^g^−1^ protein; this indicates both rumen samples possess similar biomass-degrading capacity.

Overall, highest lynx-associated enzyme activities, on gram of protein basis, were found for substrates containing β-xylose and β- and α-galactose; substrates containing α- and β-fucose, β-(methyl)glucuronide and α-glucose were hydrolyzed at lower level in both lynx samples whereas no appreciable activity was detected for *p*NP-α-xylose and *p*NP-β-arabinopyranoside (Figure 4A). For the rumen samples a more homogeneous scenario was found with similar activity levels for about nine different substrates containing β-xylose, α- and β-galactose, α- and β-glucose, β-lactose, α-arabinose (pyranose conformation) and α-rhamnose (Figure 4B). Interestingly, rumen samples did show much higher capacity (>25 fold) to hydrolyze *p*NP-β-cellobiose (indicative of hemicellulases and cellulases) as compared to the lynx samples for which this was one of the poorest substrates (Figure 4C). By contrast, rumen samples had the lowest activities for β-(methyl)glucuronides (almost below the detection limit) while they were efficiently hydrolyzed by lynx-derived proteins (from 180- to 2770-fold difference). This may agree with the fact that acyl glucuronidation is one of the major metabolic conjugation reactions of most carboxylic acid in animal tissues [71]. Additionally, whereas fucose (up to 2.9-fold) and mannose (up to 9.4-fold) substrates were slightly preferred by lynx proteins, α-glucose and malto-oligosaccharides (up to 6.0-fold) were for rumen ones (Figure 4C).

Taken together, we found enzyme activity data in good agreement with those found by *in silico* analysis of metagenome sequences, namely, an over-representation of α/β-galactosidases and β-glucuronidases and their associated activities together with a slightly lower representation of gene signatures and activities associated to lacto-N-biosidases, fucosidases and mannosidases, which comparatively are dominant in lynx gut (Figure 4C). Additionally, the low activity against *p*NP-β-cellobiose is in agreement with the under-representation of ‘presumptive’ GHF5/28/53 (hemi)cellulases in lynx metagenome as compared to cow rumen (Table S4). However, the low α-glucosidase but the high β-xylosidase activity in lynx gut protein extracts (Figure 4A) contrast with the over-representation of α-amylase and related gene signatures and the under-representation of β-xylosidase and related gene signatures in the metagenome (Table S4), respectively, which indicate that additional factors rather than gene content may contribute to the overall sugar metabolism in animal guts, i.e. lynx gut. What ever the case, the preponderance of β-xylosidase activity (even at higher level than in ruminal extracts; Figure 4C) may further suggests the ‘presumptive’ potential of the lynx gut microbial community for sugar uptake from xylose containing plant N-glycans [72]. This is of special significance since this potential metabolic capacity could not be predicted from metagenome sequencing data analysis, thus confirming the recognized hypothesis that minor enzyme components may play significant ecological role. Additionally, this result highlights the need for more complementary bio-informatics and rigorous experimental analyses to accurately ascertain the overall metabolic capacities of microbial communities, including those from distal gut.

### Conclusion

**Figure 5:**
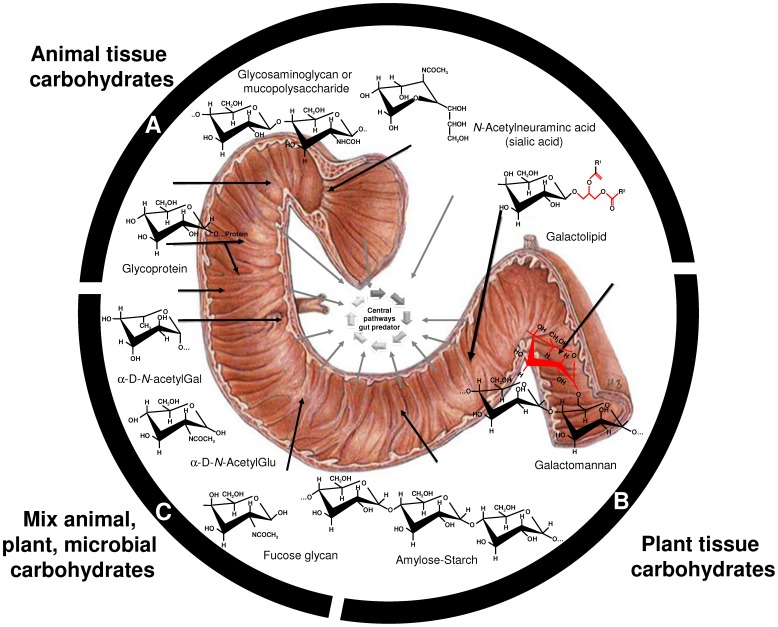
Model representing the potential sugar uptake system feeding the central lynx metabolism through the hydrolysis of carbohydrate-containing substrates derived directly from animal tissues or indirectly from plant/sugar biomass most likely derived from prey nutrition sources.

In conclusion, the metagenomic approach described in this study represents a first attempt to preliminary characterize the tremendous bacterial diversity found in wild lynx feces. According to previous studies of canine [26], [27] and feline [25] intestinal microbiota, the phylum Firmicutes was the largest phylum in lynx fecal microbial community, constituting 43.25% of the total SSU rRNA reads analyzed. The reads contained five Firmicutes classes and the majority detected was from the order Clostridiales. Bacteria affiliated with the phylum Bacteroidetes were the second most abundant group in the lynx fecal microbiota. Members of Fusobacteria were also detected in the lynx fecal sample and slightly exceeded 10% of the total reads. A similar amount (9.2% of total reads) of Fusobacteria was identified in the fecal microbial community of wolves (29), whereas this group of organisms appears to be a minor part of the intestinal ecosystem in both herbivorous and omnivorous mammals [13]. The identification of bacterial phyla such as Spirochaetes and candidate division TM7, which has not been previously detected by conventional PCR-based analysis in carnivorous mammals, will facilitate the next step in understanding the complex phylogenetic diversity of the intestinal microbial communities and will introduce previously uncharacterized bacterial phylotypes for further analysis. In addition to the bacterial diversity datasets, the collective gene-centric metagenomic findings suggest that the wild lynx contains a distinct microbiome structure with respect to sugar processing and metabolism compared to any other gut microbiome reported to date. Data likely suggest that in the lynx microbiome, together with genes encoding AqpZ aquaporin and lysosomal-like digestive enzymes most likely involved in sugar uptake from animal tissues, an almost equal contribution of enzyme sets attack the side chains of complex plant/seed polysaccharides but not the more recalcitrant primary (hemi) cellulose chains. Whereas the first set of enzymes could be directly driven by diet (wild rabbits), the second set may appears to be driven by the prey characteristics and its diet (seeds and plants). In this context, wild lynx is well known to have a monotypic diet that consists primarily of wild European rabbits that in their turn utilize seeds and plants as their principal sources of energy. Accordingly, the data presented here may suggest that both prey (primary energy supply) and its diet (secondary energy supply) may both play major roles in the nutritional ecology and evolution of the gut microbial communities of a predator. Under this scenario, gut communities from a carnivore may contain stable microbial communities that are also adapted to metabolize the sources of energy from the gut of the prey intestine (see Figure 5 for details). However, the possibility that bacteria belonging to phylum Bacteroidetes (major contributor for the sugar uptake, as revealed by binning analysis) that typically have also the metabolic capacity in their genomes to degrade a variety of complex materials, are playing a major metabolic role independently of the diet source, cannot be ruled out. In addition, the fact those bacteria are successful colonizers in the gut of lynx (Figure 1) might be also due to a starvation situation of predators in which they rely on mucus production from the host, independently of the diet source; this might also explain the high number of genes associated to the utilization of sugar components such as GalNAc, fucose and sialic acid. What ever the case, the fact that ‘presumptive’ functional microbiome structures of lynx gut differs from that of any other gut microbiome reported to date, suggest that, to some extent, prey and predator gastrointestinal microbiomes may share functional capacities for energy nutrition and that a monotypic diet may have direct effects on microbiome structures. However, the possibility of biases introduced by the different methods used to isolate DNA samples as well as the artifacts due to differences in sample size (i.e. number of reads and open reading frames) caused by the different sequencing platforms used (Sanger, Solexa, 454 pyrosequecing or Illumina), could not be ruled out when comparing gut microbiomes. Further studies are required for the characterization of the gut metagenomes of lynxes and other carnivorous animals of varying dietary regimes, locations, seasons, ages and health statuses to elucidate key factors shaping gut structure. In this context, it is known that the Canadian Lynx (*Lynx canadiensis*) has a similar monotypic diet based on snowshoe hares (*Lepus americanus*); therefore, further studies for comparing the gut microbial communities of Iberian lynx and European rabbit and Canadian lynx and snowshoe hares should enhance the understanding of the potential interactions between specific predator-prey tandems. Whatever the case, since the present investigation considered only one (for sequence analysis) and two (for activity tests) individuals, further statistical significance studies are required to ascertain the link between diet and bacterial diversity in the gut due to a monotypic diet. Finally, to the best of our knowledge this study further provided the first biochemical insights into the comparative potential of carnivorous- and ruminal-associated microbial communities to deconstruct sugars present in animal tissues and plant biomass.

## Supporting Information

Figure S1 **Location of the Guarrizas Iberian lynx reintroduction area.**
(JPG)

Figure S2 **Small subunit (SSU) rRNA length distribution identified in raw (unassembled) sequences after direct pyrosequencing of the extracted DNA from lynx fecal samples.** Sequences with a length ≥ 200 nucleotides are shown.
(JPG)

Figure S3 **Overview of the prokaryotic diversity of SSU rRNA tag sequences extracted from the lynx distal gut pyrosequences.**
(JPG)

Figure S4 **A neighbor-joining tree of the proteobacterial SSU rRNA gene sequences representing the largest clostridia families affiliated with the phylum Firmicutes.**
(JPG)

Figure S5 **A neighbor-joining tree of the proteobacterial SSU rRNA gene sequences affiliated with the phylum Firmicutes.** The number of sequences in each identity cluster is specified.
(JPG)

Figure S6 **A neighbor-joining tree of non-proteobacterial SSU rRNA gene sequences.** The number of sequences in each identity cluster is specified.
(JPG)

Table S1 **General features of the lynx gut metagenome sequences.**
(DOC)

Table S2 **Complete information regarding gene prediction and the annotation and taxonomic classification of lynx gut metagenome sequences.** Panel ‘General features’ includes, total length of the metagenome, number of hypothetical, conserved hypothetical and functional conserved proteins, number of contigs, ORFs, tRNAs, RNAs, GC content and contig length distribution; Panel ‘CDS’ includes the automatic annotation of lynx gut metagenome coding sequences; Panel ‘GenomesDB Genus 1e^−05′^ includes the result of phylogenetic binning of CDSs at the genus level according to an e value of distribution of 1e^−05^; Panel ‘GenomesDB Order 1e^−05′^ includes the result of phylogenetic binning of CDSs at the order level according to an e value of distribution of 1e^−05^; Panel ‘COG 1e^−05′^ includes the number of total ORFS and associated COG and COG distribution; Panel ‘KEGG Level 3 1e^−05′^ includes the number of genes associated to particular KEGG pathways; Panel ‘KEGG Level 2 1e^−05′^ includes the number of genes associated to particular major KEGG metabolisms. Full details of the methods used for pyrosequences annotation are given in Methods.
(XLS)

Table S3 **Complete information regarding the hierarchical clustering of the lynx gut metagenome and other gut metagenomes based on functional composition.** The metagenomes used for comparative analysis are specifically shown. Panel ‘List’ includes the list of metagenomes used for comparative analysis; Panel ‘Subset 2^nd^ Class’ includes the comparative analysis of genes represented in KEGG major categories, both in total number and percentage referred to total ORFs; Panel ‘All Kegg 1e^−05^ test’includes the total number of genes coding proteins represented in KEGG pathways; Panel ‘% for Heatmap’ includes the relative percentage of total genes distributed per KEGG major categories per metagenome; Panel ‘Distances for Heatmap’ includes the comparative distance between pairs KEGG pathway-metagenome used for clustering and heat map analyses.
(XLS)

Table S4 **Presence of glycoside hydrolases (GHs) in the lynx metagenome sample compared to that found in the metagenomes of other representative herbivores and carnivores.** Panel ‘GH distribution’ includes the total number and the relative percentage of different GHs in the metagenomes as well as the putative function associated to each GH protein family (based on bibliographic records and (CAZy) database. Functional assignment of predicted genes encoding GHs and CBMs was performed via BLASTP analysis against the CAZy database. The identified sequences were manually checked and functional assignment was performed based on the biochemical functions associated to the most similar proteins belonging to a given family. Panel ‘Detailed info relevant ORFs’ includes an extensive description of the KEGG orthologs and the taxonomic affiliations of the genes that code for GHs and other relevant genes coding enzymes of interest. GHs were distributed according to their protein families. Results of the annotation pipeline shown in Table S2 were used for both KEGG orthologs and tentative taxonomic assignments. Full details of the methods used for pyrosequences annotation are given in Methods.
(XLS)
