# Supplementary figures and images for: Gene Sets for Utilization of Primary and Secondary Nutrition Supplies in the Distal Gut of Endangered Iberian Lynx
Source: PLoS One. 2012 Dec 12;7(12):e51521. doi: 10.1371/journal.pone.0051521 (PMC3520844; doi:10.1371/journal.pone.0051521)

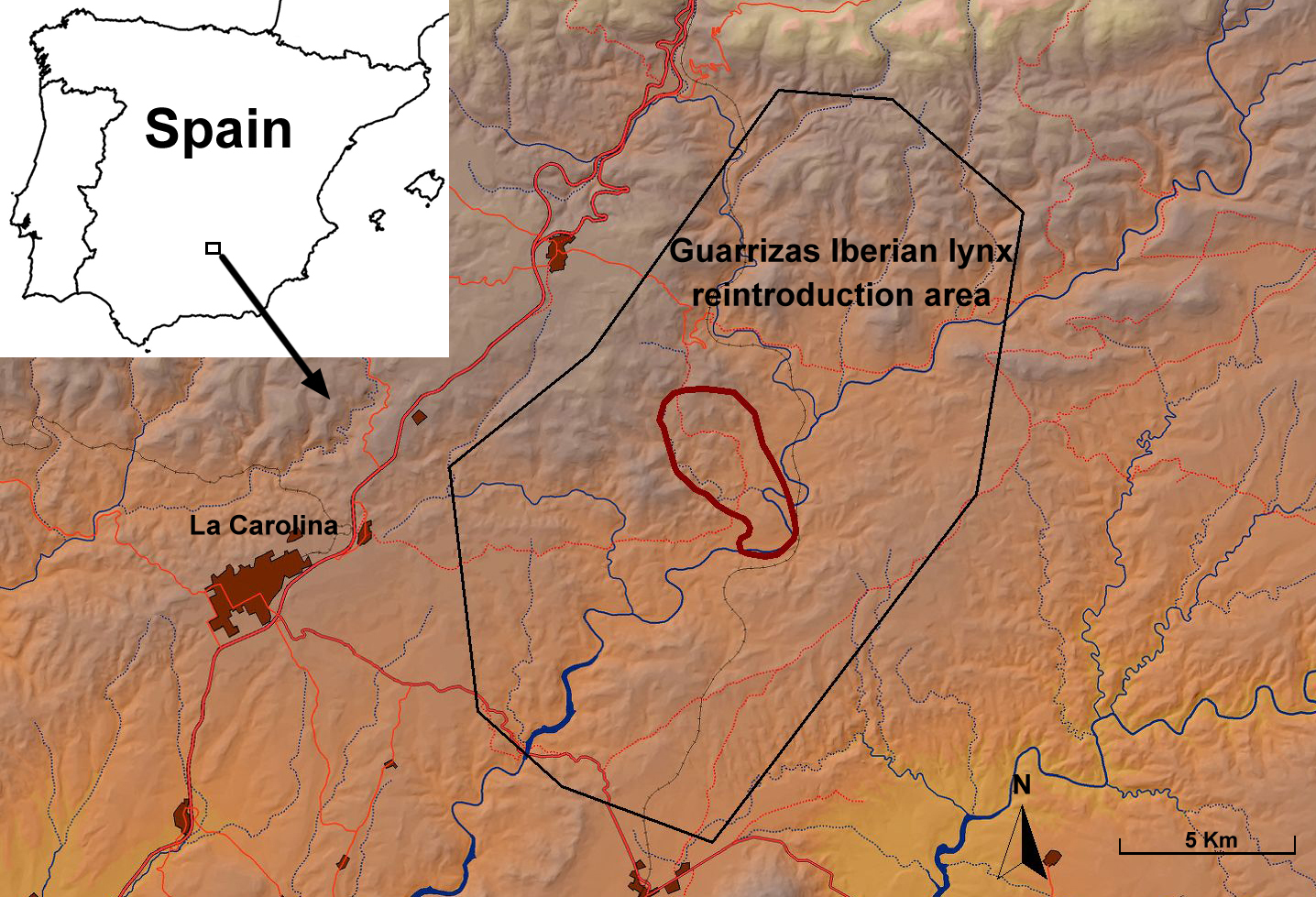

Supplement: Figure S1 — Location of the Guarrizas Iberian lynx reintroduction area. (JPG) [file pone.0051521.s001.jpg]

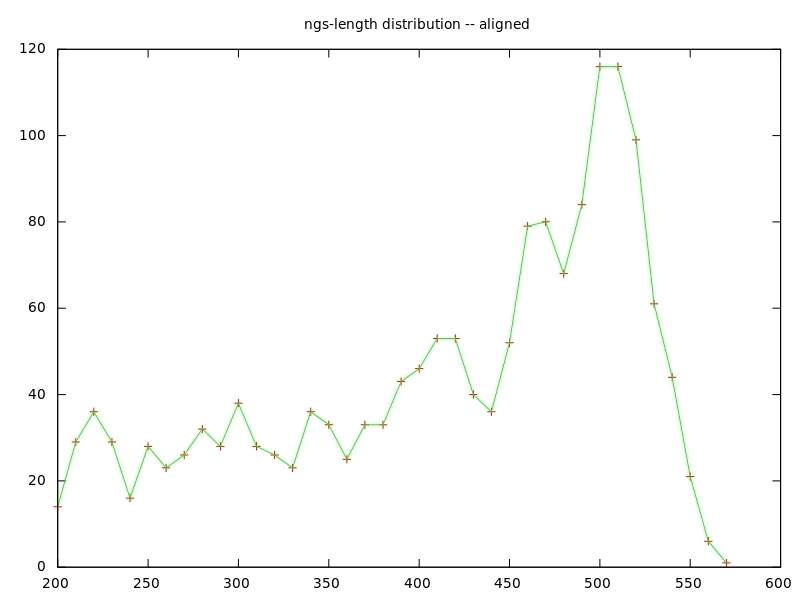

Supplement: Figure S2 — Small subunit (SSU) rRNA length distribution identified in raw (unassembled) sequences after direct pyrosequencing of the extracted DNA from lynx fecal samples. Sequences with a length ≥ 200 nucleotides are shown. (JPG) [file pone.0051521.s002.jpg]

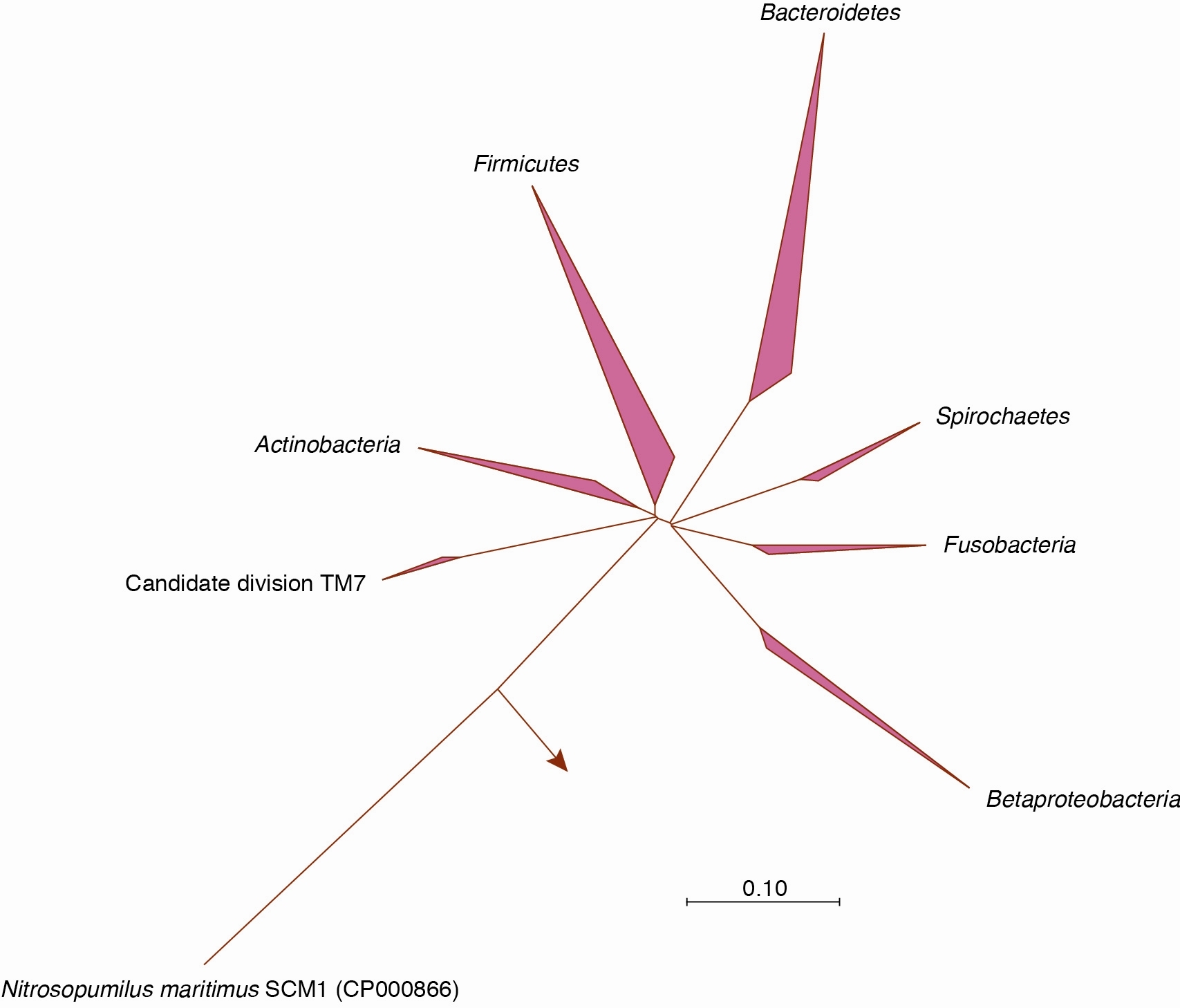

Supplement: Figure S3 — Overview of the prokaryotic diversity of SSU rRNA tag sequences extracted from the lynx distal gut pyrosequences. (JPG) [file pone.0051521.s003.jpg]

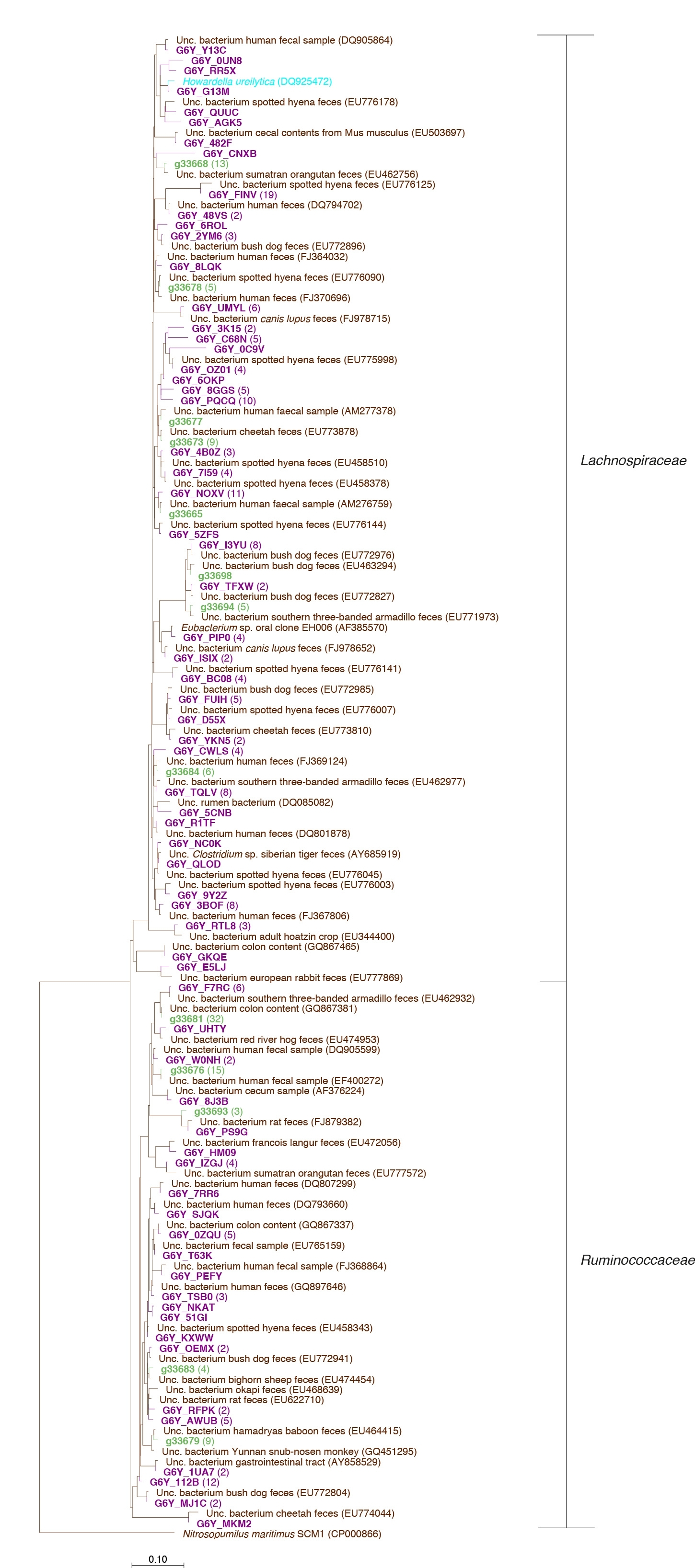

Supplement: Figure S4 — A neighbor-joining tree of the proteobacterial SSU rRNA gene sequences representing the largest clostridia families affiliated with the phylum Firmicutes. (JPG) [file pone.0051521.s004.jpg]

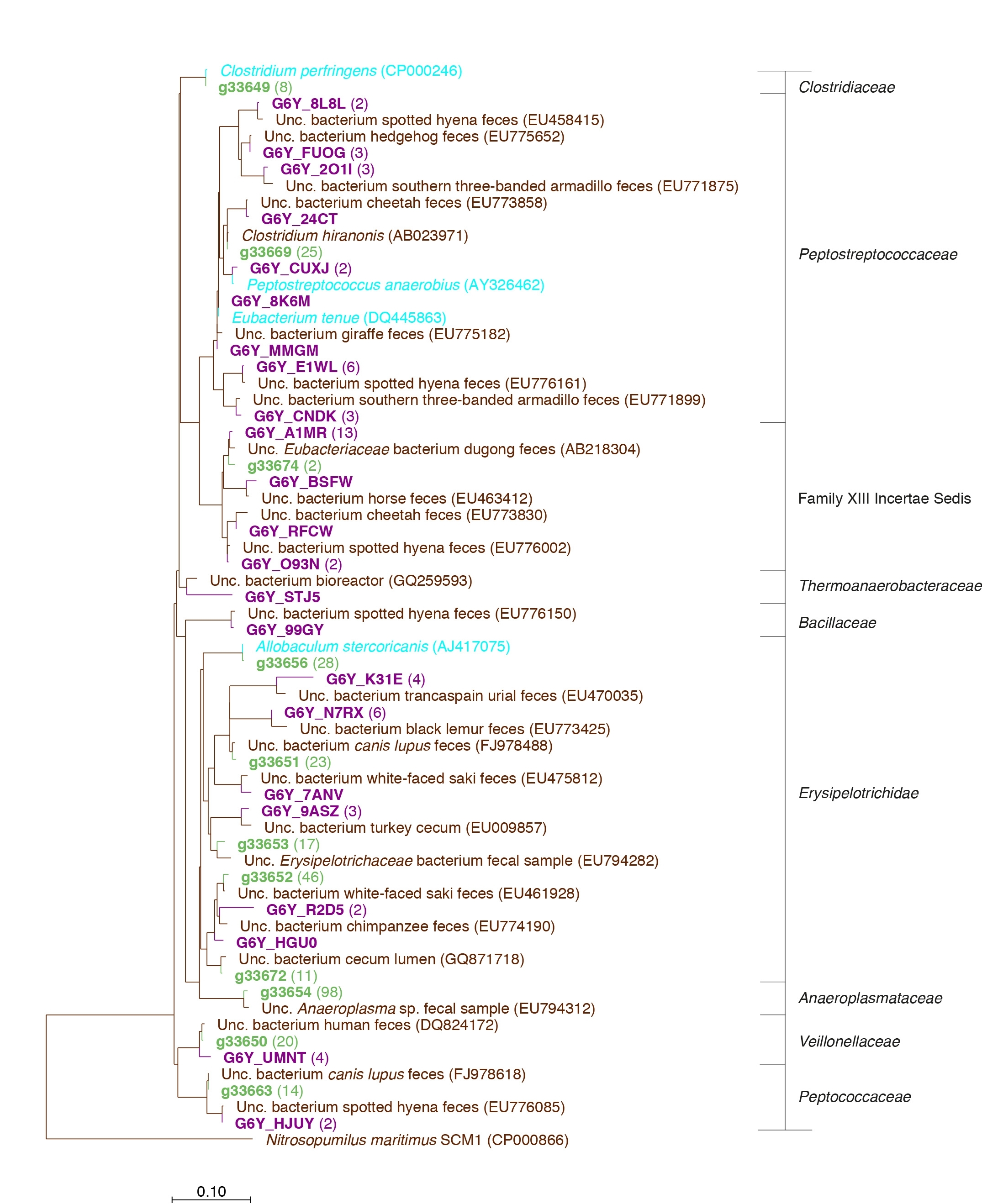

Supplement: Figure S5 — A neighbor-joining tree of the proteobacterial SSU rRNA gene sequences affiliated with the phylum Firmicutes. The number of sequences in each identity cluster is specified. (JPG) [file pone.0051521.s005.jpg]

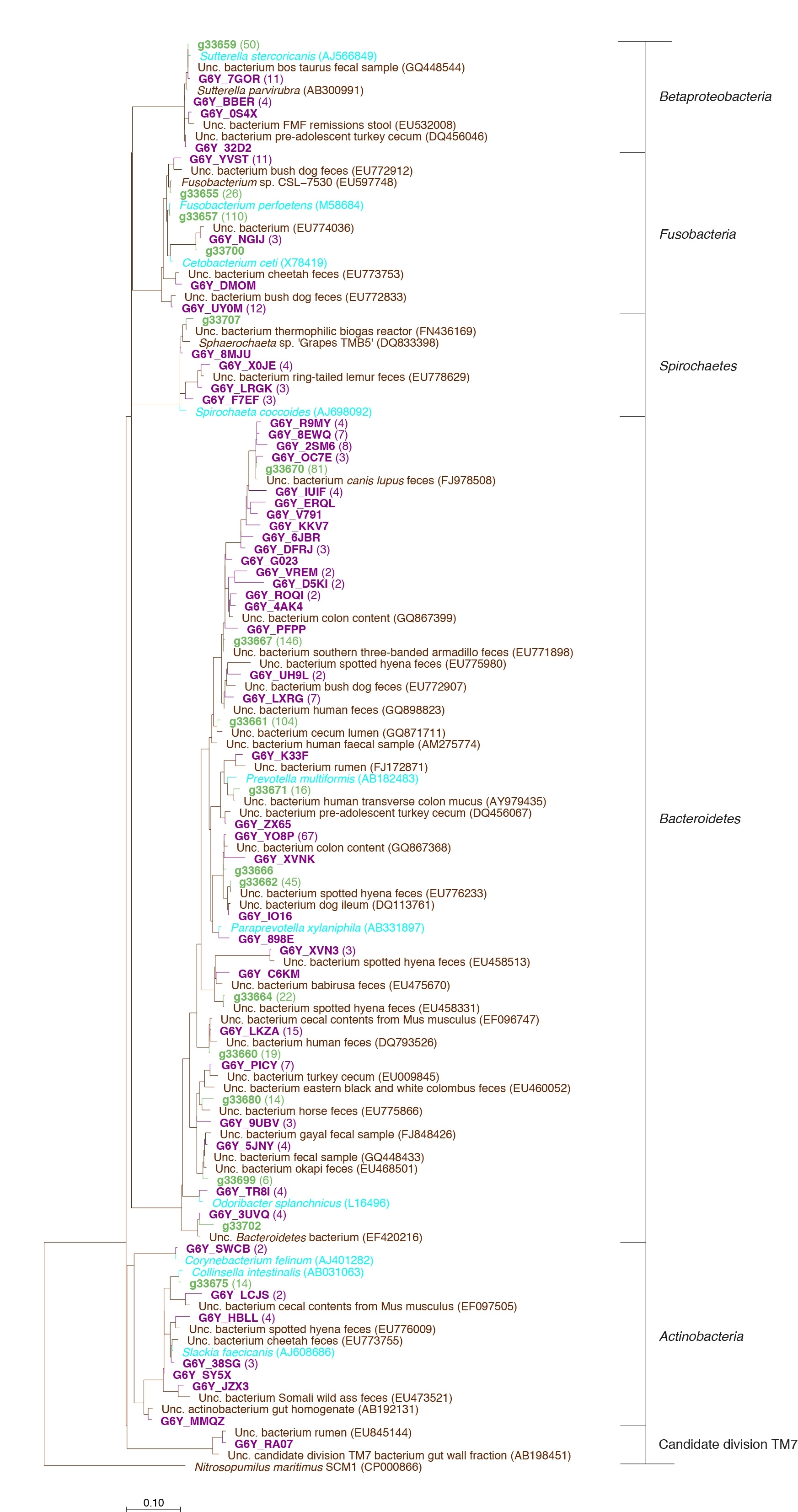

Supplement: Figure S6 — A neighbor-joining tree of non-proteobacterial SSU rRNA gene sequences. The number of sequences in each identity cluster is specified. (JPG) [file pone.0051521.s006.jpg]
