# Supplementary material for: Gene Sets for Utilization of Primary and Secondary Nutrition Supplies in the Distal Gut of Endangered Iberian Lynx
Source: PLoS One. 2012 Dec 12;7(12):e51521. doi: 10.1371/journal.pone.0051521 (PMC3520844; doi:10.1371/journal.pone.0051521)
Supplement: Table S1 — General features of the lynx gut metagenome sequences. (DOC) [file pone.0051521.s007.doc]

**Table S1 General features of the metagenome of the lynx feces**

| Parameter | Processed* | Submitted |
| --- | --- | --- |
| Size (bp) | 18336115 | 18005334 |
| Contigs | 11068 | 9707 |
| Average contig size (bps) | 1657 | 1856 |
| Average GC content (%) | 47.74 | 47.77 |
| Protein-coding genes (CDS) | 23780 | 22214 |
| Average CDS size (bp) | 609 | 635 |
| tRNAs | 533 | 510 |
| rRNAs | 251 | 195 |
| ORFs with predicted function | 11465 | 11196 |
| Hypothetical | 7052 | 6485 |
| Conserved hypothetical | 5263 | 4533 |
| Assigned to COGs | 9642 | 9434 |
| Assigned to KEGG | 12725 | 12407 |

* data is available on request
